# Age-period-cohort analysis of smoking prevalence among young adults in Korea

**DOI:** 10.4178/epih.e2016010

**Published:** 2016-03-19

**Authors:** Yong Ho Jee, Sung-il Cho

**Affiliations:** Graduate School of Public Health, Seoul National University, Seoul, Korea

**Keywords:** Cigarette smoking, Prevalence, Age, Period, Cohort effect

## Abstract

**OBJECTIVES:**

Smoking prevalence among Korean men in their thirties is substantially high (approximately 50%). An in-depth analysis of smoking trends among young adults in their twenties is necessary to devise antismoking policies for the next 10 years. This study aimed to identify the contributions of age, period, and birth cohort effects on smoking prevalence in young adults.

**METHODS:**

Subjects comprised 181,136 adults (83,947 men: 46.3%; 97,189 women: 53.7%) aged 19 to 30 years from the 2008-2013 Korea Community Health Survey. Smoking prevalence adjusted with reference to the 2008 population was applied to the age-period-cohort (APC) model to identify the independent effects of each factor.

**RESULTS:**

For men, smoking prevalence rapidly escalated among subjects aged 19 to 22 years and slowed down among those aged 23 to 30 years, declined during 2008 to 2010 but stabilized during 2011 to 2013, and declined in birth cohorts prior to 1988 but stabilized in subjects born after 1988. However, in APC models, smoking prevalence increased with age in the 1988 to 1991 birth cohort. In this birth cohort, smoking prevalence at age 19 to 20 years was approximately 24% but increased to 40% when the subjects turned 23 to 24 years. For women, smoking prevalence was too low to generate consistent results.

**CONCLUSIONS:**

Over the past six years and in recent birth cohorts, smoking prevalence in adults aged 19 to 30 years has declined and is stable. Smoking prevalence should be more closely followed as it remains susceptible to an increase depending on antismoking policies or social conditions.

## INTRODUCTION

Smoking is known as a major cause of various chronic illnesses, such as cancer or cardiovascular and neurovascular diseases [1]. Despite the fact that similar findings were reported in Korea as well [2,3], smoking prevalence among Koreans is still considerably high.

In 2013, 37% of men in their twenties, 54.5% of men in their thirties, and 48.0% of men in their forties were smokers in Korea, with the highest smoking prevalence among men in their forties [4]. In consideration of the fact that men in their twenties currently will enter the thirties and forties age group in 10 years, this study would be meaningful in that it could provide basic data for predicting smoking patterns of men in their thirties and forties in the future by analyzing smoking patterns among men currently in their twenties.

The 2013 Korea Health and Nutrition Examination Survey was not a reliable reference for the analysis of age-specific smoking patterns because it examined 5,338 persons, with <760 persons aged <30 years. On the other hand, the Korea Community Health Survey (KCHS) has been conducted since 2008 on adults >19 years of age. This survey studied 228,781 persons in 2013 [5], of which 27,696 were in the 19 to 30-year age group. According to the 2008-2013 KCHS, the overall smoking prevalence is on the decline, although there are regional differences in this trend [6]. Unfortunately, the current young adult group is more prone to increased smoking prevalence than any other age group owing to substantial stress arising from fierce competition in the job market and prevalent social inequity [7]. Despite such significance, however, most domestic and foreign studies analyzing smoking trends have usually focused on teenagers or adults [6,8], with only a few analyzing young adults (aged 19 to 30 years).

In addition, most studies on smoking prevalence were cross-sectional studies [6,8], which were inadequate for analyzing age-specific and birth cohort–specific smoking prevalence [9-11]. The age-period-cohort (APC) model has been widely used to address this problem [12-14]. The APC model in particular has been used more in the past for analyses of trends in long-term mortalities and incidences of chronic diseases [12], and recently it has been used to analyze smoking trends as well as the effects of smoking on death rates [15-17].

By assessing the independent effects of age, period, and birth cohorts on the changes in smoking prevalence among young adults aged 19 to 30 years, this study sought to provide preliminary data for developing antismoking policies and predicting prospective smoking prevalence in adults aged ≥30 years.

Despite several reports shedding light on age-specific and period-specific smoking trends, not many studies have controlled for confounding effects of these factors and examined their independent associations with smoking trends. Therefore, this study established the following hypotheses for testing:

- Age effect: Smoking prevalence will increase as age increases.
- Period effect: Smoking prevalence will decrease with increasing recency of period.
- Birth cohort effect: Smoking prevalence will be lower among younger cohorts.

## MATERIALS AND METHODS

### Subjects

The Korea Centers for Disease Control and Prevention implemented the KCHS in 2007 in 20 cities, districts, and precincts. The project was expanded in 2008 to encompass 247 cities, districts, and precincts nationwide and is conducted every year. This study examined 181,136 adults (83,947 men: 46.3%; 97,189 women: 53.7%) aged 19 to 30 years in the KCHS data spanning 2008 to 2013. More specifically, the subjects comprised 29,212 persons in 2008, 34,192 persons in 2009, 31,946 persons in 2010, 29,558 persons in 2011, 28,537 persons in 2012, and 27,696 persons in 2013 (Appendix 1).

### Study variables

To identify smoking patterns in young adults using the APC model for smoking prevalence, we extracted the following variables from the yearly survey data: period, age, gender, number of subjects, number of current smokers, and weighted values. “Current smoker” was defined as persons who had answered “I smoke every day” to the item “Do you currently smoke?”

### Statistical analysis

#### Analysis of weighted smoking prevalence

To examine the changes of age-specific, period- specific, and birth cohort–specific smoking prevalence for both men and women subjects of the KCHS, we requested and obtained KCHS raw data for years 2008 to 2013. We applied a two-year interval for dividing age groups from 19 to 30 (19-20, 21-22, 23-24, 25-26, 27-28, 29-30) for each period (year), and the period was also divided into three groups (2008-2009, 2010-2011, 2012-2013). Birth cohorts were divided into eight groups (1978-1979, 1980-1981, 1982-1983, 1984-1985, 1986-1987, 1988-1989, 1990-1991, 1992-1993) to analyze smoking prevalence for each cohort (Appendix 2). We computed smoking prevalence via PROC SURVEYFREQ after weighting the samples for each region and enumeration district. Furthermore, we used age-adjusted smoking prevalence based on the 2008 national population to eliminate the differences caused by changes in age distribution.

#### Age-period-cohort analysis

The changes in smoking prevalence from 2008 to 2013 were examined in terms of age, period-cohort, age-period, and age-cohort effect. Furthermore, the independent effects of each factor on smoking prevalence were estimated via an APC analysis. The problem of identification caused by the linearity among age, period, and cohort (age+cohort=period) [13,14] was solved by using the intrinsic estimator (IE) method. The fitness of the APC model with different combinations of age, period, and cohort was analyzed using the Akaike information criteria and differences in deviance values. For the IE, the apc_ie package of Stata version 13.1 (StataCorp., College Station, TX, USA) was used. All analyses were performed separately for men and women subjects. All statistical analyses were performed using SAS version 9.3 (SAS Institute Inc., Cary. NC, USA) and Stata 13.1, with me a significance level of p<0.05.

## RESULTS

Table 1 shows the gender-specific, age-specific, and period–specific smoking prevalence in subjects. In men, smoking prevalence increased with age in all periods. However, in all period groups, smoking prevalence declined with increasing recency of period. Women did not show a consistent smoking prevalence.

**Figure 1.**
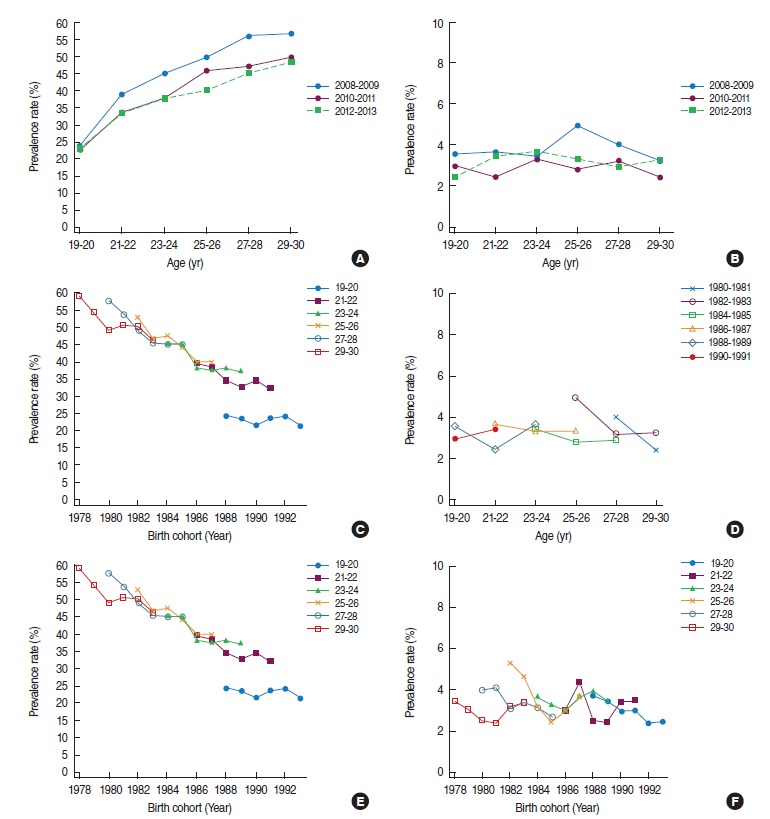
Age-period-cohort effect of age-specific smoking prevalence in 2008 to 2013 in men and women. Age-specific smoking prevalence by period in men (A) and women (B), age-specific smoking prevalence by birth cohort in men (C) and women (D), and birth cohort-smoking prevalence by age in men (E) and women (F).

Figure 1 shows the age, period, and birth cohort effects on changes in smoking prevalence. Figure 1A is an illustration of age-specific smoking prevalence in men by period. Age-specific smoking prevalence was higher in 2008 to 2009 than in 2012 to 2013. Figure 1C shows the age-specific smoking prevalence by birth cohort. Overall, smoking prevalence declined or remained unchanged with increasing age in older birth cohorts. However, in the 1988 to 1991 birth cohort, smoking prevalence continued to increase with increasing age. In other words, smoking prevalence for 1988 to 1991 cohorts at age 19 to 20 years was approximately 24%, but it rose to approximately 40% at age 23 to 24 years.

Figure 1D is an illustration of age-specific smoking prevalence by birth cohorts in women. Unlike men, women were not associated with distinct trends per cohort but, overall, smoking prevalence declined with increasing age in older cohorts, most prominently in the 1980 to 1981 cohort. There were some decreasing trends in the 1982 to 1983 cohort as well, but no such trend from the 1986 cohort onward.

Figure 1E shows birth cohort-specific smoking prevalence by age intervals in men. In all age groups except the 19 to 20-year group, smoking prevalence declined with increasingly younger cohorts. However, the rate of decline slowed in recent cohorts. There was no consistent birth cohort–specific smoking prevalence in women.

**Figure 2.**
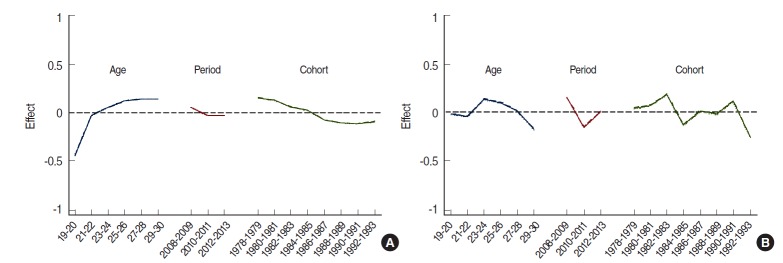
Age-period-cohort analysis of smoking prevalence in Korean men (A) and women (B).

Table 2 shows the results of analyses of other models for smoking prevalence. Of these models, the APC model best explained smoking prevalence of both men and women (Table 2, Appendix 3). Figure 2 is a schematic diagram of the APC model in Table 2. For men, the independent effects of each factor were as follows. First, smoking prevalence rapidly escalated from age 19 to 22 years while the rate of increase slowed from age 23 to 30 years. Second, smoking prevalence slowly declined from 2008 to 2010 but was stable from 2011 to 2013. Third, smoking prevalence declined in birth cohorts prior to 1988, but it was stable in those born after 1988. However, there was no consistency in smoking prevalence among women.

## DISCUSSION

This study identified the independent effects of age, period, and cohort on the changes of smoking prevalence in adults aged 19 to 30 years via the APC model. The data for the study were collected from the 2008-2013 KCHS. Smoking prevalence increased with increasing age and declined with increasing recency of period and birth cohort, but currently remains stable. One notable trend was that in the 1988 to 1991 cohort, smoking prevalence continued to increase with age. Unlike men, women were associated with relatively inconsistent smoking patterns.

Most existing studies analyzing smoking prevalence focused on teenagers [6,8], adults [6], women [18], and the elderly [19], but rarely examined young adults. The excessive focus on teenagers and women is presumed to be due to the societal implication of increasingly younger ages of smoking initiation and the health-related implication that smoking is detrimental to pregnancy and women’s health. Furthermore, adults and the elderly were considered important subjects of the study as the effects of smoking could be confirmed in these age groups. Young adults are somewhat outside of this circle of research interest. However, as this study has stressed, young adults are a very important age group in terms of developing anti-smoking policies because young adults will eventually age to replace the current adults in their thirties and forties, which is the age group with the highest smoking prevalence. From this standpoint, the rising smoking prevalence in a recent birth cohort (i.e., 1988 to 1991) should not be overlooked. Policymakers should take note of this fact and concentrate efforts on devising policies that would prohibit further escalation of smoking prevalence in young adults, which would be a critical step toward reducing smoking prevalence in adults overall.

In most studies, cross-sectional data were used to analyze smoking prevalence. Such study designs are limited in identifying age-specific smoking prevalence as well as birth cohort effects. However, recently, several studies have attempted to perform APC analysis of smoking prevalence by combining cross-sectional data for several years [20-22]. Veday [20] performed APC analysis of smoking prevalence in accordance with education level and gender and explained smoking prevalence in terms of social changes associated with each birth cohort and the theory of diffusion of innovations. Moreover, Veday [20] suggested that smoking prevalence declined with increasing education level via intercohort diffusion. Chen et al. [21] reported that 1969 to 1985 birth cohorts were associated with a high smoking prevalence while relatively younger cohorts (those born after 1985) had a lower smoking prevalence. They explained that this phenomenon was presumed to be due to the anti-smoking campaigns targeting teenagers and the increase in the number of tobacco advertisements targeting teenagers in 1980 to 1997.

Existing studies have suggested that in general, with the exception of a few cohorts, more recent cohorts had lower smoking prevalence; in contrast, Carreras & Gorini [22] found that smoking prevalence rose for both men and women in more recent cohorts. In this study using KCHS data, smoking prevalence declined with increasing recency of cohorts overall, but in one recent cohort, smoking prevalence actually increased as the cohorts aged. This signifies that there is a possibility that smoking prevalence in Korean young adults may decline or increase in the future. In this study, smoking prevalence in women was inconsistent and did not show particularly distinct trends. This might be a result of low smoking rates in women and low accuracy and reliability of smoking prevalence data for women, but further research is required to draw definite conclusions.

As previously mentioned, smoking prevalence among young adults in this study increased with increasing age, but decreased with increasing recency of period and birth cohort. The declining smoking prevalence in Korean young adults may be explained with the following reasons. Young adults have shown high smoking prevalence due to the teenager-targeting marketing strategies of tobacco companies and excruciating stress arising from fierce competition in college admissions and the job market but, nonetheless, smoking prevalence declined. This may be a result of tough anti-smoking policies, one of which was the increase in cigarette prices [23].

Meanwhile, there are possible explanations for the increasing smoking prevalence in Korean young adults. We found that smoking prevalence in the 1988 to 1991 birth cohorts was still on the rise with increasing age during the period of this study. That is, smoking prevalence among the 1988 to 1991 cohorts at age 19 to 20 years was approximately 24%, but it rose to approximately 40% at age 23 to 24 years, signifying that it is still not clear whether smoking prevalence in this cohort will continue to increase as the cohorts age.

Foreign studies on the factors that cause an increase of smoking in young adults classified the factors into personal risk factors and social and environmental effects. Ellickson et al. [24] reported that some personal risk factors for smoking are parents’ low education level, low high school grades, and younger ages in grade cohorts. Emmons et al. [25] conducted a multi-level analysis on college students in the US and found that smokers were more associated with the use of illegal drugs, such as marijuana; excessive drinking; and promiscuous sexual relationships. Weschler et al. [26] revealed that college students considered smoking as a means to alleviate stress and lose weight.

One of the social factors that have contributed to smoking in young adults is the strategic youth target marketing by tobaco industires. Worldwide movement of tough smoking regulations for teenagers, which has directed the attention of tobacco companies toward young adults, driving these companies to engage in aggressive marketing to this age group [27]. In the late 1980s, US tobacco companies promoted their products by handing out samples in places that young adults frequent, such as bars or clubs. In fact, an internal document of a US tobacco company revealed that tobacco companies strategically selected young adults as their targets for promotions linked to drinking or clubbing cultures [28].

There are several advantages to this study. First, this study used representative data from the KCHS. Second, in terms of methodology, it employed the APC model to analyze long-term smoking prevalence. In fact, studies that used APC models mostly examined the independent effects of these factors in long-term mortalities or disease incidences [12,27]. This method allows researchers to analyze the age effect; period effect, defined as the social and environmental effect on everyone in a particular period; and birth cohort effect, which only specific birth cohorts experience, as separate risk factors. However, separating these three effects is challenged by the statistical linearity among them [13,14]. We solved this identification problem by using the IE [12].

The 1989 and 1990 birth cohorts overlapped in the period of this study. Even if the period was divided into two-year intervals, such as 2008 to 2009, 2010 to 2011, and 2012 to 2013, there would be overlapping cohorts. The optimal method would be to apply one-year intervals for age and period with each of the birth cohorts, but this would significantly reduce sample size, which in turn would induce large variations in smoking prevalence. However, although the present study divided periods into one-year and two-year intervals in the APC model to analyze the independent effects of each factor, we did not find significant differences in the results. Second, the validity of the smoking prevalence data also has limitations in that self-report measurements are always accompanied by a possibility of underreporting, which undermines the accuracy and reliability of the data. Overall, we expect that these limitations will be resolved to a certain extent in future studies with longer follow-up periods and additional research on the validity and reliability of smoking prevalence data.

In conclusion, the findings of smoking prevalence in Korean young adults based on the KCHS data for the past six years indicate that smoking prevalence among young adults aged 19 to 30 years declined with increasing recency of period and birth cohorts but is currently stable. Conversely, in one young cohort, smoking prevalence continued to increase with age. It is important to carefully observe variations in smoking prevalence, as it is still vulnerable to an increase depending on antismoking policies or social conditions.

## Figures and Tables

**Table 1.** Smoking prevalence according to gender, age, and period: 2008-2013 Korea Community Health Survey

Gender	Age (yr)	Period									p for trend
		2008-2009			2010-2011			2012-2013			
		n	%	SE	n	%	SE	n	%	SE	
Men	19-20	2,971	23.8	0.8	3,437	22.7	0.7	3,826	22.8	0.7	0.96
	21-22	3,642	39.0	0.8	3,813	33.5	0.8	3,987	33.3	0.7	<0.001
	23-24	4,589	45.0	0.7	4,388	37.7	0.7	4,226	37.6	0.7	<0.001
	25-26	5,699	49.9	0.7	4,710	45.8	0.7	4,149	40.0	0.8	<0.001
	27-28	6,522	55.8	0.6	5,450	47.2	0.7	4,391	45.1	0.8	<0.001
	29-30	6,407	56.6	0.6	6,411	49.7	0.6	5,329	48.3	0.7	<0.001
Women	19-20	4,357	3.6	0.3	5,170	3.0	0.2	5,498	2.4	0.2	0.07
	21-22	4,406	3.7	0.3	4,439	2.4	0.2	4,808	3.4	0.3	0.05
	23-24	4,788	3.4	0.3	4,593	3.0	0.2	4,496	3.7	0.3	0.65
	25-26	5,990	4.9	0.3	5,095	2.8	0.2	4,445	3.3	0.3	0.001
	27-28	6,979	4.0	0.2	6,372	3.2	0.2	6,167	2.9	0.2	0.05
	29-30	7,054	3.2	0.2	7,611	2.4	0.2	7,054	3.2	0.2	0.14

SE, standard error.

**Table 2.** Goodness of fit of APC for smoking prevalence among Korean young adults

Gender	Model	AIC	DEV	df	Δ DEV	Δ df
Men	APC	149.9	2,398.8	4	0.0	0
	AC	385.5	6,641.2	5	4,242.4	1
	AP	386.7	6,673.4	10	4,274.6	6
	PC	3,019.1	54,053.1	8	51,654.3	4
	Age	3,969.6	71,175.5	12	68,776.7	8
Women	APC	63.7	893.0	4	0.0	0
	AC	499.7	8,744.2	5	7,851.2	1
	AP	511.6	8,968.5	10	8,075.5	5
	PC	301.8	5,187.2	8	4,294.2	4
	Age	1,165.3	20,739.0	12	19,846.0	8

APC, age-period-cohort model; AIC, Akaike information criteria; DEV, deviance; df, degree of freedom; AC, age-cohort model; AP, age-period model; PC, period-cohort model.

## Appendix 1.

Number of men and women subjects according to age and period used in the study, 2008-2013 Korea Community Health Survey data

## Appendix 2.

Birth cohorts per age and period

## Appendix 3.

Age-period-cohort model for smoking prevalence among Korean young adults
