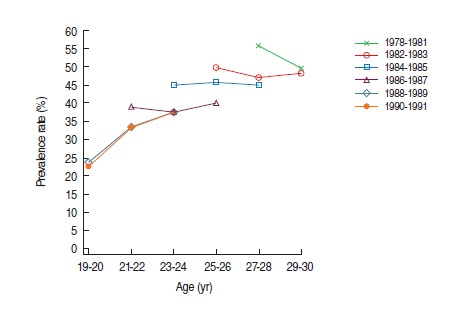# Age-period-cohort analysis of smoking prevalence among young adults in Korea

**DOI:** 10.4178/epih.2016029

**Published:** 2016-07-08

**Authors:** Yong Ho Jee, Sung-il Cho

**Affiliations:** Graduate School of Public Health, Seoul National University, Seoul, Korea

This article was initially published in the Epidemiology and Health 2016;38:e2016010, with an error in Figure 1C that duplicated Figure 1E.

The authors would like to correct Figure 1C as below.