# Supplementary material for: Age-period-cohort analysis of smoking prevalence among young adults in Korea
Source: Epidemiol Health. 2016 Mar 19;38:e2016010. doi: 10.4178/epih.e2016010 (PMC4877516; doi:10.4178/epih.e2016010)
Supplement: Supplementary file 1 [file epih-38-e2016010-app1.pdf]

Appendix 1. Number of men and women subjects according to age and period used in the study, 2008-2013 Korea Community Health Survey data

	Age (yr)	Period					
		2008	2009	2010	2011	2012	2013
Men	19	763	1,060	1,039	1,132	1,130	1,233
	20	486	662	671	595	725	738
	21	674	868	866	868	887	938
	22	941	1,159	1,030	1,049	1,108	1,054
	23	999	1,202	1,158	1,043	1,016	1,092
	24	1,148	1,240	1,162	1,025	1,064	1,054
	25	1,272	1,452	1,188	1,107	1,046	1,011
	26	1,406	1,569	1,312	1,103	1,073	1,019
	27	1,554	1,732	1,437	1,127	1,080	1,037
	28	1,512	1,724	1,574	1,312	1,218	1,056
	29	1,452	1,852	1,606	1,473	1,301	1,202
	30	1,485	1,618	1,763	1,569	1,443	1,383
Women	19	943	1,294	1,317	1,360	1,426	1,426
	20	918	1,202	1,187	1,306	1,310	1,336
	21	958	1,163	1,181	1,075	1,181	1,285
	22	1,024	1,261	1,096	1,087	1,211	1,131
	23	1,029	1,263	1,204	1,063	1,120	1,147
	24	1,193	1,303	1,220	1,106	1,168	1,061
	25	1,335	1,493	1,253	1,171	1,077	1,123
	26	1,552	1,610	1,384	1,287	1,094	1,151
	27	1,666	1,836	1,552	1,348	1,226	1,128
	28	1,624	1,853	1,827	1,645	1,342	1,215
	29	1,625	1,935	1,893	1,763	1,567	1,357
	30	1,653	1,841	2,016	1,939	1,724	1,519
Total		29,212	34,192	31,936	29,553	28,537	27,696

Appendix 2. Birth cohorts per age and period

Age (yr)	Period					
	2008	2009	2010	2011	2012	2013
19-20	1988-1989	1989-1990	1990-1991	1991-1992	1992-1993	1993-1994
21-22	1986-1987	1987-1988	1988-1989	1989-1990	1990-1991	1991-1992
23-24	1984-1985	1985-1986	1986-1987	1987-1988	1988-1989	1989-1990
25-26	1982-1983	1983-1984	1984-1985	1985-1986	1986-1987	1987-1988
27-28	1980-1981	1981-1982	1982-1983	1983-1984	1984-1985	1985-1986
29-30	1978-1979	1979-1980	1980-1981	1981-1982	1982-1983	1983-1984
