# Supplementary material for: Age-period-cohort analysis of smoking prevalence among young adults in Korea
Source: Epidemiol Health. 2016 Mar 19;38:e2016010. doi: 10.4178/epih.e2016010 (PMC4877516; doi:10.4178/epih.e2016010)
Supplement: Supplementary file 3 [file epih-38-e2016010-app3.pdf]

Appendix 3. Age-period-cohort model for smoking prevalence among Korean young adults

	Factor	Interval	Regression coefficient	Standard error	p-value
Men	Age	Age_19-20	-0.4392	0.0011	<0.001
		Age_21-22	-0.0253	0.0009	<0.001
		Age_23-24	0.0558	0.0008	<0.001
		Age_25-26	0.1218	0.0007	<0.001
		Age_27-28	0.1443	0.0006	<0.001
		Age_29-30	0.1425	0.0006	<0.001
	Period	Period_2008-09	0.0546	0.0004	<0.001
		Period_2010-11	-0.0295	0.0005	<0.001
		Period_2012-13	-0.0269	0.0010	<0.001
	Cohort	Cohort_1978-79	0.1563	0.0007	<0.001
		Cohort_1980-81	0.1287	0.0007	<0.001
		Cohort_1982-83	0.0627	0.0007	<0.001
		Cohort_1984-85	0.0259	0.0009	<0.001
		Cohort_1986-87	-0.0720	0.0010	<0.001
		Cohort_1988-89	-0.1020	0.0009	<0.001
		Cohort_1990-91	-0.1107	0.0010	<0.001
		Cohort_1992-93	-0.0089	0.0019	<0.001
Women	Age	Age_19-20	-0.0197	0.0030	<0.001
		Age_21-22	-0.0440	0.0028	<0.001
		Age_23-24	0.1316	0.0030	<0.001
		Age_25-26	0.0994	0.0028	<0.001
		Age_27-28	0.0092	0.0025	<0.001
		Age_29-30	-0.1764	0.0027	<0.001
	Period	Period_2008-09	0.1479	0.0016	<0.001
		Period_2010-11	-0.1533	0.0018	<0.001
		Period_2012-13	0.0054	0.0017	0.002
	Cohort	Cohort_1978-79	0.0392	0.0039	<0.001
		Cohort_1980-81	0.0698	0.0030	<0.001
		Cohort_1982-83	0.1810	0.0027	<0.001
		Cohort_1984-85	-0.1335	0.0034	<0.001
		Cohort_1986-87	0.0113	0.0033	0.001
		Cohort_1988-89	-0.2093	0.0029	<0.001
		Cohort_1990-91	0.1126	0.0056	<0.001
		Cohort_1992-93	-0.0295	0.0019	<0.001
